# The role of macroinvertebrates for conservation of freshwater systems

**DOI:** 10.1002/ece3.3101

**Published:** 2017-06-15

**Authors:** Carolina Nieto, Ximena M.C. Ovando, Rafael Loyola, Andrea Izquierdo, Fátima Romero, Carlos Molineri, José Rodríguez, Paola Rueda Martín, Hugo Fernández, Verónica Manzo, María José Miranda

**Affiliations:** ^1^ Instituto de Biodiversidad Neotropical (IBN) CONICET‐UNT San Miguel de Tucumán Tucumán Argentina; ^2^ Facultad de Ciencias Naturales e I.M.L. San Miguel de Tucumán Tucumán Argentina; ^3^ Laboratório de Malacologia Límnica e Terrestre Departamento de Zoologia Instituto de Biologia Roberto Alcantara Gomes Universidade do Estado do Rio de Janeiro (UERJ), Maracanã Rio de Janeiro Brazil; ^4^ Laboratório de Biogeografia da Conservação Departamento de Ecologia Universidade Federal de Goiás Goiânia Goiás Brazil; ^5^ Instituto de Ecología Regional (IER) CONICET‐UNT Yerba Buena Tucumán Argentina; ^6^ Fundación Miguel Lillo San Miguel de Tucumán Tucumán Argentina

**Keywords:** connectivity, conservation planning, invertebrates, South America, spatial prioritization, species distribution models, watersheds, zonation

## Abstract

Freshwater ecosystems are the most threatened ecosystems worldwide. Argentinian‐protected areas have been established mainly to protect vertebrates and plants in terrestrial ecosystems. In order to create a comprehensive biodiverse conservation plan, it is crucial to integrate both aquatic and terrestrial systems and to include macroinvertebrates. Here, we address this topic by proposing priority areas of conservation including invertebrates, aquatic ecosystems, and their connectivity and land uses. Location: Northwest of Argentina. We modeled the ecological niches of different taxa of macroinvertebrates such as Coleoptera, Ephemeroptera, Hemiptera, Megaloptera, Lepidoptera, Odonata, Plecoptera, Trichoptera, Acari, and Mollusca. Based on these models, we analyzed the contribution of currently established protected areas in the conservation of the aquatic biodiversity and we propose a spatial prioritization taking into account possible conflict regarding different land uses. Our analysis units were the real watersheds, to which were added longitudinal connectivity up and down the rivers. A total of 132 species were modeled in the priority area analyses. The analysis 1 showed that only an insignificant percentage of the macroinvertebrates distribution is within the protected areas in the North West of Argentina. The analyses 2 and 3 recovered similar values of protection for the macroinvertebrate species. The upper part of Bermejo, Salí‐Dulce, San Francisco, and the Upper part of Juramento basins were identified as priority areas of conservation. The aquatic ecosystems need special protection and 10% or even as much as 17% of land conservation is insufficient for species of macroinvertebrates. In turn the protected areas need to combine the aquatic and terrestrial systems and need to include macroinvertebrates as a key group to sustain the biodiversity. In many cases, the land uses are in conflict with the conservation of biodiversity; however, it is possible to apply the connectivity of the watersheds and create multiple‐use modules.

## 1 INTRODUCTION

Freshwater systems are recognized as one of the main suppliers of ecosystem services (MEA 2005), in terms of economics value, culture, science, and education. Historically, human settlements and industries have been established near rivers because different human activities, especially agriculture, make extensive use of water. However, the undesirable consequence of having human activities close by freshwater ecosystems is that these activities contribute to pollution, eutrophication, and erosion, not only in the rivers but also in the aquifer (Vörösmarty et al., 2010). Consequently, although these ecosystems harbor a unique biodiversity (Schröter et al., 2005), along with their provided services, they are the most threatened ecosystems worldwide (MEA 2005).

The main reason for the extreme vulnerability of freshwater ecosystems is probably because the disproportionate richness of inland waters as a habitat for plants and animals. The freshwater habitats cover about 0.8% of the Earth′s surface but they support 9.5% of all animal species described (Turak et al., 2017). Furthermore, one‐third of all vertebrate species inhabit fresh waters (Dudgeon et al., 2006).

The functionality of ecosystems can be affected in different ways through the loss of biodiversity. Biodiversity loss reduces the efficiency of ecological communities to capturing essential resources, producing biomass, decomposing, and recycling essential nutrients. Similarly biodiversity loss reduces the ability to stabilize ecosystem functions throughout time (Cardinale et al., 2012). Establishing protected areas to prevent such loss of biodiversity and ecosystem services has long been a strategy adopted by NGOs and governments (Loucks, Ricketts, Naidoo, Lamoreux, & Hoekstra, 2008; but see Brooks et al., 2004; Le Saout et al., 2013; Watson, Dudley, Segan, & Hockings, 2014 and Nori et al., 2015) and is especially important in regions with intensive human land use (Dobrovolski, Diniz‐Filho, Loyola, & Júnior, 2011; Dobrovolski, Loyola, Da Fonseca, Diniz‐Filho, & Araújo, 2014; Luck, 2007; Nori et al., 2015).

Argentina has 444 protected areas placed in different categories, covering nearly 12% of the country′s surface (Sistema Federal de Áreas Protegidas, SIFAP). This is still not enough for the level of coverage recommended by the Secretary of the Convention on Biological Diversity (CBD) which proposed at least 10% of terrestrial and inland waters should be conserved by 2010 (Hirsch, 2010) and 17% by 2020 (Secretariat of CBD, 2014) in order to reduce the rate of biodiversity loss. The inclusion of species within protected areas is essential to effectively predict future extinction rates (Pimm & Joppa, 2015).

One problem of the Argentinian‐protected areas is that they only focus on the protection of plants (Ortega‐Baes et al., 2012) and vertebrates (Arzamendia & Giraudo, 2004; Corbalán, Tognelli, Scolaro, & Roig‐Juñent, 2011; Nori et al., 2013; Tabeni, Bender, & Ojeda, 2004; Tognelli, Abba, Bender, & Seitz, 2011). Invertebrates are not considered when planning or considering protected areas, even if their need has been showed (Chehébar et al., 2013). Another problem Argentina faces is that even though several protected areas have been created, most of these are located in regions undergoing a decreasing intensity of land use, failing to protect the eco‐regions most threatened by current land use trends (Izquierdo & Grau, 2009).

**Figure 1:**
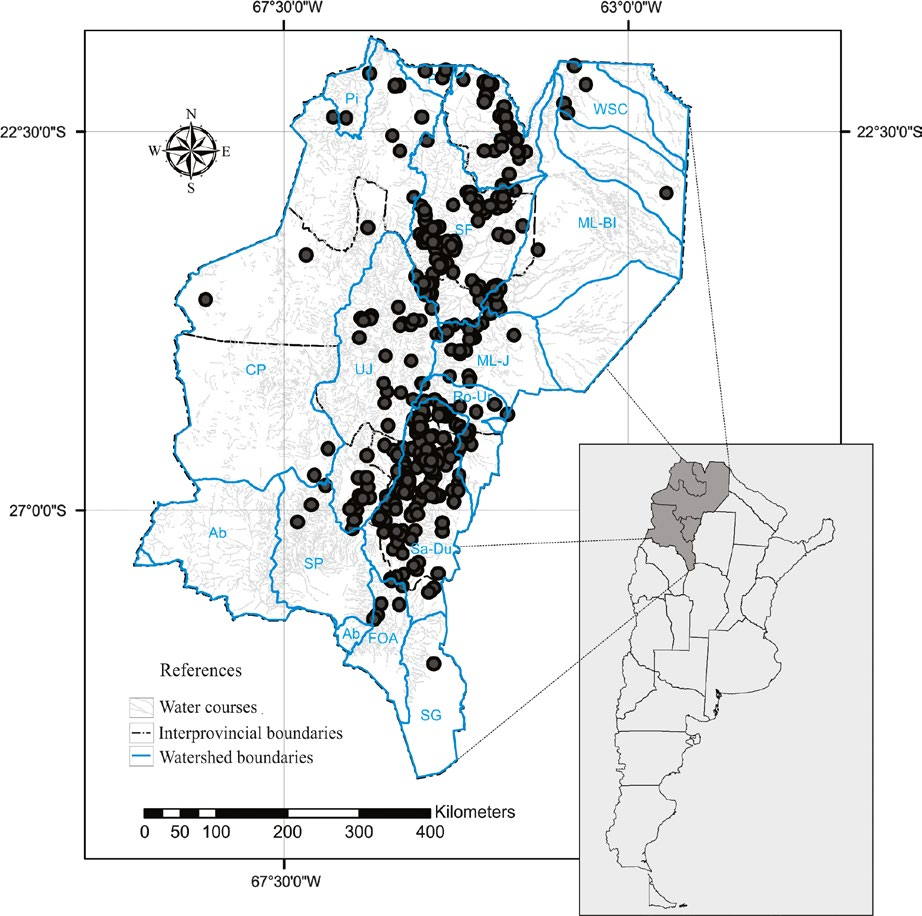
Study area (Northwest of Argentina) showing in detail water courses and watersheds boundaries. (PI, Pilcomayo river; BS, Bermejo Superior or Upper part of Bermejo; SF, San Francisco river; ML‐BI, middle‐lower part of Bermejo; CP, Cuenca Cerrada de la Puna; UJ, Río Juramento Superior or , Upper part of Juramento; ML‐J, Juramento Medio‐Inferior; Ro‐Ur, Rosario Horcones‐Urueña river; Sa‐Du, Salí‐Dulce river; Ab, Abaucan; SP, Salar de Pipanaco; FOA, Falda Oriental de Ambato; SG, Salinas Grandes, WSC, without significant contribution watershed).

Land use change is the main component of regional environmental change (e.g., Geist & Lambin, 2002; Laurence et al., 2002; Vitousek, Mooney, Lubchenko, & Melillo, 1997). Previous studies have shown that in the last decades, human population and land use trends varied among ecological areas within the Northwest region of Argentina (Izquierdo & Grau, 2009). While human population became concentrated in urban areas, mainly localized near rivers, agricultural production has become concentrated in the areas more suitable for modern agriculture, such as Chaco Dry Forest, while marginal agriculture areas and extensive grazing are decreasing. One of the most threatened systems in the northwest of Argentina is the freshwater ecosystems (Figure 1). Deforestation and clearing of forests for agriculture (mainly sugar cane, kidney bean, and soybean) along with the establishment of industries which use water indiscriminately are a serious issue for the provision of water not only in the region itself but also in neighboring areas (Grau et al., 2007). These critical conditions have always been difficult to handle, regulate, and control as land owners allege unwanted possible economic and social consequences (Brown, 2009).

Over the last 30 years, the science of systematic conservation planning has developed concepts, methods, and tools to catalyze agreements focused on biodiversity conservation when faced with conflicting interests (Moilanen, Wilson, & Possingham, 2009). Given that the northwest Argentinean freshwater ecosystems are under severe threat, that macroinvertebrates inhabiting these ecosystems are critical for their functioning due to their role in nutrients cycling, processing an enormous amount of organic matter and as food source for many organisms. However, conservation planning for invertebrates is still pending worldwide.

Species distribution models (SDMs) have been used to predict the present and future species distributions in other groups (Faleiro, Machado, & Loyola, 2013). These analyses have been extensively applied with freshwater species using the worldclim variables (Campos et al., 2014; Giovanelli, Haddad, & Alexandrino, 2008; Kumar et al., 2009; Vasconcelos, Rodriguez, & Hawkins, 2012). These models examine the role of specific environmental variables affecting the distribution of species at various spatial scales and can help to determine appropriate management actions (Kumar et al., 2009). The ecosystem services and biological process can only be sustained by the identification of conservation units, which includes biodiversity patterns and connection between the different ecosystems (Margules & Sarkar, 2007).

Our project presents the first spatial plan for the conservation of freshwater ecosystems for this region including the macroinvertebrates as a fundamental protection target. In order to achieve this plan, we first modeled the ecological niche of more than 120 macroinvertebrates species. We evaluated the contribution of currently established protected areas in the region to conserve these species. The priority areas for conservation were designed to avoid conflicts with competing land uses including the longitudinal upstream–downstream connections along the basins. This connectivity is the key factor in the distribution of aquatic communities and is crucial for the conservation planning of these systems.

## 2 METHODS

### 2.1 Study area

The study was carried out in the northwest region of Argentina (NOA) and included four provinces: Jujuy, Salta, Tucumán, and Catamarca (Figure 1). This region extends from 20°00′–30°05′S and 62°21′–69°25′W with a total area of 333.833 km^2^. The region is subtropical with well‐defined wet and dry periods. Summer rainfall accounts for 70–90% of the annual total and is followed by a dry winter season (Paolini, Villalba, & Grau, 2005). The geography of this region is strongly affected by the relief, which has an impact on the weather, the vegetation, and hydrography (Sesma, Guido, & Puchulu, 1998). It is located at an altitude range between 300 and almost 7,000 m.a.s.l., which creates different landscape units or ecoregions (Brown & Pacheco, 2005) such as

- High Andean (Altos Andes): The land use is marginal, characterized by extensive grazing (Izquierdo & Grau, 2009).
- High elevation plateaus (Puna): The land use is characterized by extensive grazing and small family‐managed agriculture fields (Izquierdo & Grau, 2009).
- Middle‐elevation deserts (Monte de Sierras y Bolsones): The land use includes extensive grazing and irrigated modern agriculture (Izquierdo & Grau, 2009).
- Foggy grasslands (Partizales de neblina): The land use is characterized by extensive grazing with some horticultural development in the valley. It also included some minor townships located in this area (Izquierdo & Grau, 2009).
- Humid forests (Yungas): On the slopes, the land use is dominated by extensive grazing, and selective logging, the main agriculture and the largest urban centers have developed in the foothills (Izquierdo & Grau, 2009).
- Dry forests (Chaco): The land use includes grazing, some irrigated agriculture, rain fed agriculture, and about 10 major cities (Izquierdo & Grau, 2009).
- There are two different types of river basin in the region. In the Andean section, the basins have an endorreic origin; the small streams converge in salinity depressions forming lagoons or meadows (Paoli, Elena, Mosciaro, Ledesma, & Noé, 2014). The other type of basin starts in the mountains and passes through cities and provides water all year round. Examples of this type are the following: Bermejo, Juramento, and Salí‐Dulce river basins. These characteristics result in many industries discharging their effluents into the rivers; for that reason, the latter is the second most polluted basin in Argentina.

### 2.2 Data collection

The 2,425 field records from 170 species gathered were analyzed from the repository of localities of the Aquatic Macroinvertebrates Database, administered by the Instituto de Biodiversidad Neotropical (IBN), CONICET‐UNT, Argentina. Most of the data points were the result of field trips along more than 15 years in the NOA. Intensive fieldwork was carried out by taxonomists collecting different rivers and habitats in this area. To obtain comparable measurement of the diversity of macroinvertebrates, the sampling effort was standardized in time. The time window spanned 30 min at each site, collecting as many specimens as possible. Additional sources include specialized literature and systematic studies performed on material from other collections (Instituto Fundación Miguel Lillo, Tucumán, Argentina; National Museum of Natural History, Washington, USA).

**Figure 2:**
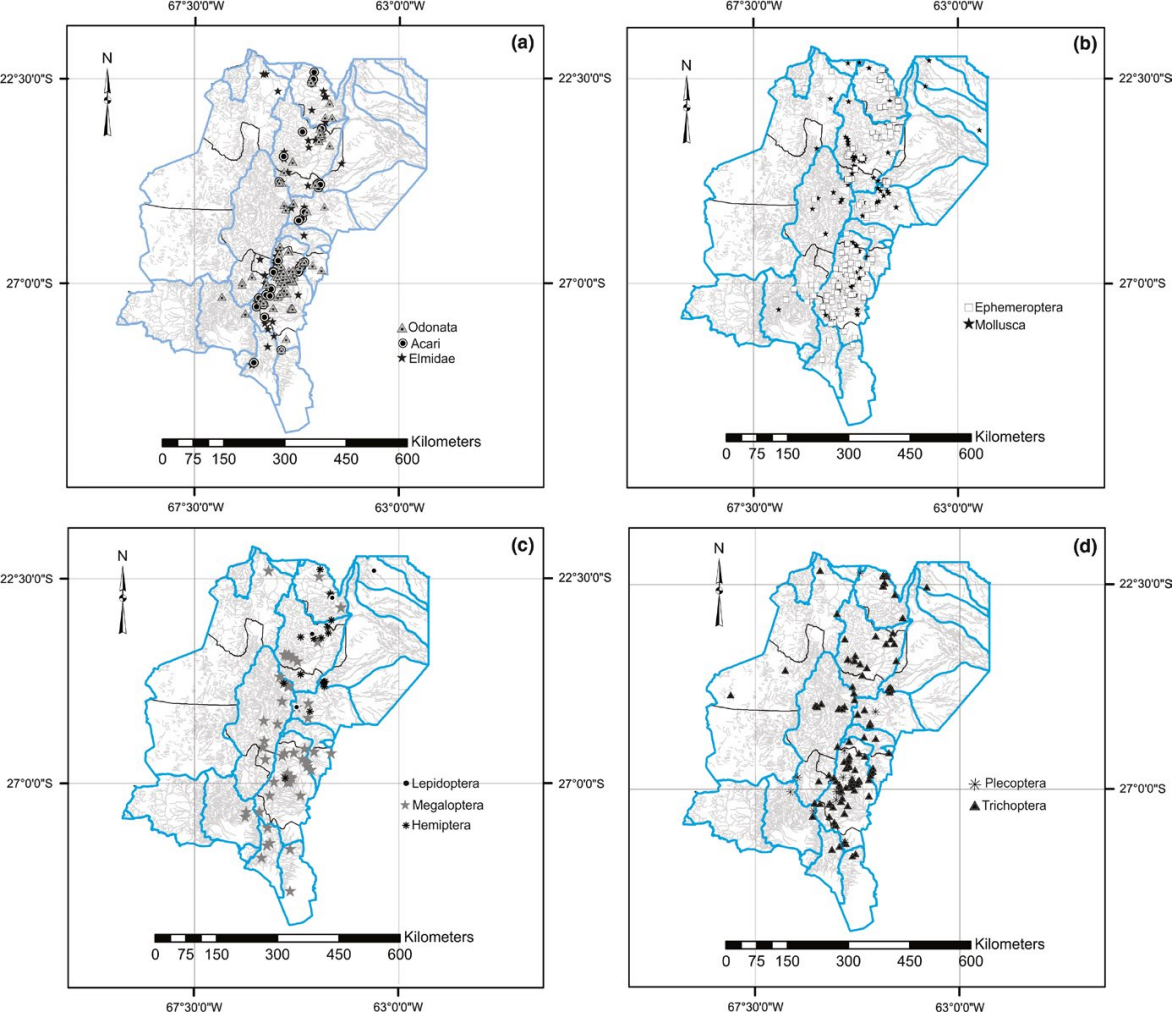
Maps showing the known distribution for Macroinvertebrates taxa in the study area. (a) Elmidae, Acari, and Odonata. (b) Ephemeroptera and Mollusca. (c) Lepidoptera, Megaloptera and Hemiptera. (d) Plecoptera and Trichoptera

Larvae and adults of macroinvertebrates were collected using standardized methods with kicknet, D‐frame nets, and light traps. All the specimens were conserved in 96% ethyl alcohol. To identify the specimens, adults (mollusks) and mature larvae (insects) were selected and dissected. Dissected parts of the specimens were mounted on microscope slides with Canada balsam or glycerin. Either a stereomicroscope or an Olympus BX‐51 microscope was used to study the specimens. Hjarding, Tolley, and Burgess (2014) stressed that there is an enormous difference in the degree of determination precision and taxonomic accuracy between specialist‐determined material and massive databases uploaded without specialist supervision. Consequently, each species and geographical data were re‐checked prior to carrying out distributional analyses. The insect orders included in the analysis were as follows: Coleoptera (12 species), Ephemeroptera (35 species), Hemiptera (six species), Lepidoptera (one species), Megaloptera (for species), Odonata (39 species), Plecoptera (two species), Trichoptera (33 species) (Appendix S1). Acari (22 species), Bivalvia (four species), and Gastropoda (12 species) were included as representatives of the noninsect macroinvertebrate fauna (Figure 2).

The data were analyzed in Diva‐Gis 7.5 (Hijmans et al., 2012) to evaluate the distribution of the species included in the analysis. Species distributions were plotted and overlapped with layers with different types of information such as political subdivisions in the northwest of Argentina__s protected areas and land uses. Layers corresponding to administrative areas of Argentina were obtained from DIVA resources (http://www.diva-gis.org/gdata). The shape files of protected areas, and indigenous people, were obtained from Fundación Proyungas (http://siga.proyungas.org.ar/recursos). Maps corresponding to rivers/streams/lagoons were obtained from Instituto Geográfico Nacional (IGN, http://www.ign.gob.ar/sig). Land use planning and native forest spatial planning were obtained from Secretaría de Ambiente y Desarrollo Sustentable de la Nación (SAyDS). River basins and subbasins were identified using a supervised classification of Landsat TM images with 30 × 30 pixels and LT1 preprocessing level. The Level 1T (L1T) data product provides systematic radiometric accuracy and geometric accuracy by incorporating ground control points and employs a digital elevation model (DEM) for topographic accuracy (http://landsat.usgs.gov/descriptions_for_the_levels_of_processing. php).

### 2.3 Ecological niche models

For ecological niche models (ENMs), the maximum entropy method was applied using the software package MaxEnt version 3.3.3k (Phillips, Anderson, & Schapire, 2006). MaxEnt is a program for modeling species distributions from presence‐only species records and combines biological data of species occurrence with environmental characteristics so as to estimate the suitable distribution over the study area (Elith et al., 2006, 2011). Default parameters of MaxEnt algorithm were used, including a maximum of 500 iterations with a convergence threshold of 0.00001 and 10,000 randomly chosen background localities. Following recommendations by Yackulic et al. (2013), the presence‐only data were chosen as they offer a better opportunity to learn about species distributions and their relationships to environmental covariates (Yackulic et al., 2013). At the same time, the data set used as collected according to a structured sampling design. The logistic output format was chosen for model values because it provides an estimated probability of presence between 0 (unsuitable for species presence) and 1 (highly suitable for species presence). The logistic output is simply a logistic transformation of the raw output, which indicates a relative probability of occurrence. In fact, it could be a real probability of occurrence if the prevalence of the species in your training data was exactly equal to the prevalence of the species in reality across the hole study but this is hard to verify as the true prevalence is generally unknown.

Twenty‐two variables were used in the present models (Appendix S2): 19 bioclimatic variables derived from monthly min/max temperature and rainfall data considered as average annual trends for the period 1950–2000, according to the WorldClim data base (Hijmans, Cameron, Parra, Jones, & Jarvis, 2005, http://www.worldclim.org/) with a spatial resolution of 30 s (1 km). The layer with different types of soil was obtained from INTA (Instituto Nacional de Tecnología Agropecuaria), the layer of rivers/streams from IGN (Instituto Geográfico Nacional, República Argentina) and the layer of altitude was obtained from Landsat TM images as previously explained. Species with four or more records of occurrence available were modeled in MaxEnt, and two groups of species were arranged according to their number of records: species with 4–10 records (considered a low number) and species with ≥11 records (considered a high number). In the group with a low number of records, the validation of the models was performed using the Jackknife validation approach (Pearson, Raxworthy, Nakamura, & Townsend Peterson, 2007), following the criterion explained in Corbalán et al. (2011) and Rinnhofer et al. (2012). The number of iterative runs for Jackknife validation was set as equal to the number of records available. The models were tested using the ValueCompute software (Pearson et al., 2007), and only species with significant models were included in the reserve‐selection analysis. In the group with a high number of records (≥11 records), 100 replicates were run and a random test percentage option was used with 75% of presence records randomly selected to generate models, while the remaining 25% was used to test them. For each model performed, the performance was assessed using the method of the area under the curve (AUC) of the receiver operating characteristic (ROC). The AUC represents the probability for the model to score a presence site (test locality) higher than a random background site (Elith et al., 2006; Phillips et al., 2006). AUC values can range between 0.5 (no predictability) and 1.0 (perfect prediction) (Jarnevich & Reynolds, 2011). Following Elith et al. (2006) and Loo, Mac Nally, and Lake (2007), for groups with high number of records, those models that have an AUC value >0.75 have a useful amount of discrimination.

### 2.4 Conservation prioritization analysis

Zonation v 3.1 (Moilanen et al., 2005, 2012) was used to identify which of the macroinvertebrate taxon distributional areas were priority for conservation. Possible conflicts between the priority areas found and the different land uses (urban areas, deforestation areas, and native forest spatial planning) were analyzed.

Zonation operates using large grids of environmental adjustment data as input files, providing a direct link between the ENMs software and spatial conservation prioritization (Moilanen et al., 2005).

It identifies areas which would ensure the survival of a given species by focusing on maximizing the habitat size, quality, and connectivity simultaneously for many conservation features, such as species, genes, habitat types, or ecosystem services (Moilanen et al., 2005; Taberlet et al., 2012). Zonation produces a hierarchical prioritization of the landscape based on the conservation value of sites (cells), accounting for complementarity. The algorithm consists of removing the least valuable cells for conservation from the landscape while minimizing marginal loss of conservation value, accounting for connectivity needs and priorities assigned to biodiversity features, such as species and land cover types, among others. In this process, the least useful sites receive the lowest ranks (close to 0) and areas most valuable for biodiversity receive the highest ranks (close to 1). This ranking is nested, meaning that the top 1% is within the top 2%, which is within the top 5% and so on. It can be visualized as a priority rank map with different colors indicating rank values (Lehtomäki & Moilanen, 2013).

In this study, Core‐area Zonation procedure was chosen as a removal rule; it produces solutions with species that occur at higher densities, but with less overlap between species, and emphasizes locations with high occurrence for each species retaining as much of the core distribution of the species as possible (Moilanen & Kujala, 2008; Moilanen et al., 2012). For each Zonation analysis performed, the predicted distributions of only those macroinvertebrate species that were validated as their primary input were used. Species of special interest (SSI) include the endemic species with fewer than four geographical records which were not modeled in Maxent, together with those not statistically significant with the Jackknife validation approach.

The analyses were performed using directed freshwater connectivity, which implies adding connectivity up and down the river (Moilanen Leathwick, & Elith, 2008). Seventy sub‐basins were treated as planning units.

Three analyses were performed:

The first assessed the existing protected areas by analyzing the percentage of macroinvertebrate distributions within these areas (A1). In this case, the protected area layer was used as a “mask” (Moilanen et al., 2012). This layer included 37 areas which are under some kind of protection, 15 of which are province reserves, seven national parks, five provincial parks, three natural monuments, and five reserves without categories.

The second considered included only the distribution of the species (A2) and the sub‐basins as unit areas. The priority areas were analyzed following the resolution of CBD, in 2010 at least 10% of inland water must be conserved (Hirsch, 2010) and until 2020 at least 17% (Secretariat of CBD, 2014). This analysis represents only the “ideal situation” necessary to identify which areas have high priority for macroinvertebrate conservation.

The third one, called “Balancing Alternative land uses” (A3) proposed by Moilanen et al. (2012), was used to analyze the potential conflicts between priority areas for conservation and land uses such as agriculture, cattle rearing, and urban areas.

A positive weight of one was given to each species, while conflicting land use features were assigned a negative weight of 46. The sum of negative weight and positive weight was 0. This analysis showed a land priority ranking; in which biodiversity is kept in the upper fraction, whereas areas suitable for land uses are in the lower section. In the Zonation analyses, land uses that represent negative weights are preferentially removed early in the planning process according to establish by Moilanen et al. (2012). The priority areas were analyzed using the previous parameters, 10% and 17%, respectively, and the sub‐basins were used as unit area.

## 3 RESULTS

From the total number of species analyzed, 132 were modeled for their ecological niche (Appendix S1). From those, 119 species were included in the conservation prioritization analyses due to their high AUC scores. The rest were excluded from the analyses due to low AUC scores or because their models did not obtain *p *< .05 according to the Jackknife validation. Endemic species that were not modeled, together with those not found statistically significant using the Jackknife validation approach (in a total 30 species), were categorized in the prioritization analysis as species of special interest (SSI). The bioclimatic variables that contributed the most to the MaxEnt model were as follows: precipitation of the wettest month (BIO 13) and mean diurnal range (BIO 2), followed by annual precipitation (BIO 12) and altitude (BIO 14). In contrast, the variables that contribute the least to the models were different variables related to the temperature (BIO 1, 4‐5, 7‐11).

**Figure 3:**
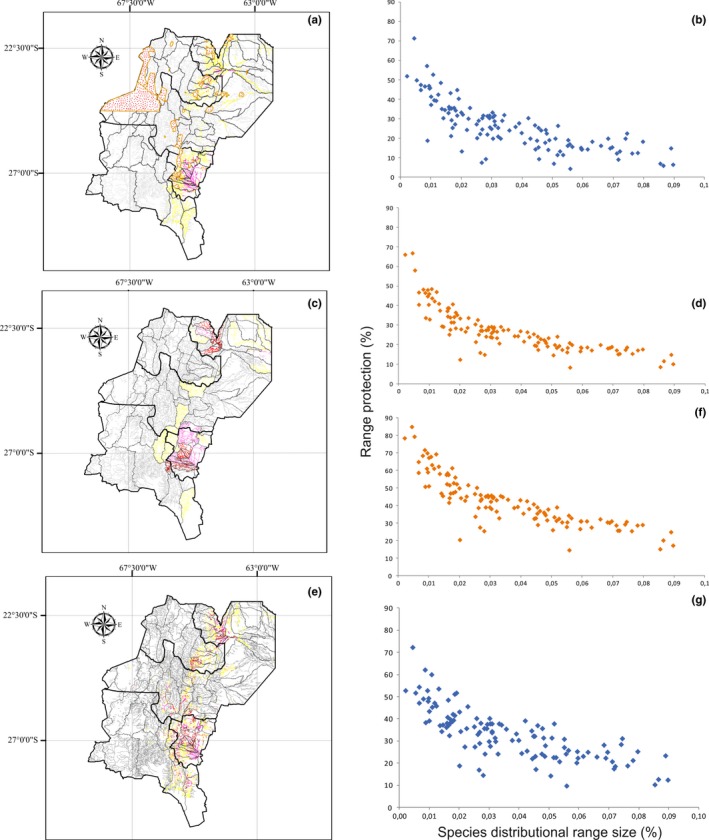
Priority conservation areas obtained by Zonation. (a) Map showing the existing protected areas (as polygons) and priority areas for conservation (A1). (b) Map showing priority areas considering only the distribution of the species (A2). (c) Map showing the priority areas of conservation for Macroinvertebrates with balancing alternative land uses considered (A3)

The first analysis (A1) showed that only a small percentage of the average distribution range of macroinvertebrates species (only 0.009%) is within the current protected areas (Figure 3a). The priority areas obtained in this analysis are located in north–south direction in this study area including the sub‐basins: Colorado river (upper part of Bermejo river basin), Bermejo river (middle‐lower Bermejo river basin), San Franscisco river, Ledesma, and Negro river (San Francisco river basin) and almost all the sub‐basins of the Salí‐Dulce basin.

Results obtained from the second analysis (A2) (Figure 3b) showed that the sub‐basins of the upper part of Bermejo river and upper basin of the Salí‐Dulce river had the top rank priority conservation for the macroinvertebrate fauna. In the Bermejo river basin, two sub‐basins had top values: the first sub‐basin formed by the tributaries Colorado, Pescado, and inferior‐Grande de Tarija rivers and the second sub‐basin with the LosToldos‐Lipeo river tributaries (in red, reddish brown, and pink). On the other hand, the Salí‐Dulce basin had the following sub‐basins with top values: Lules, Seco, Famaillá, Aranillas and Romano, Balderrama, Gastona, Chico, and Marapa river.

**Figure 4:**
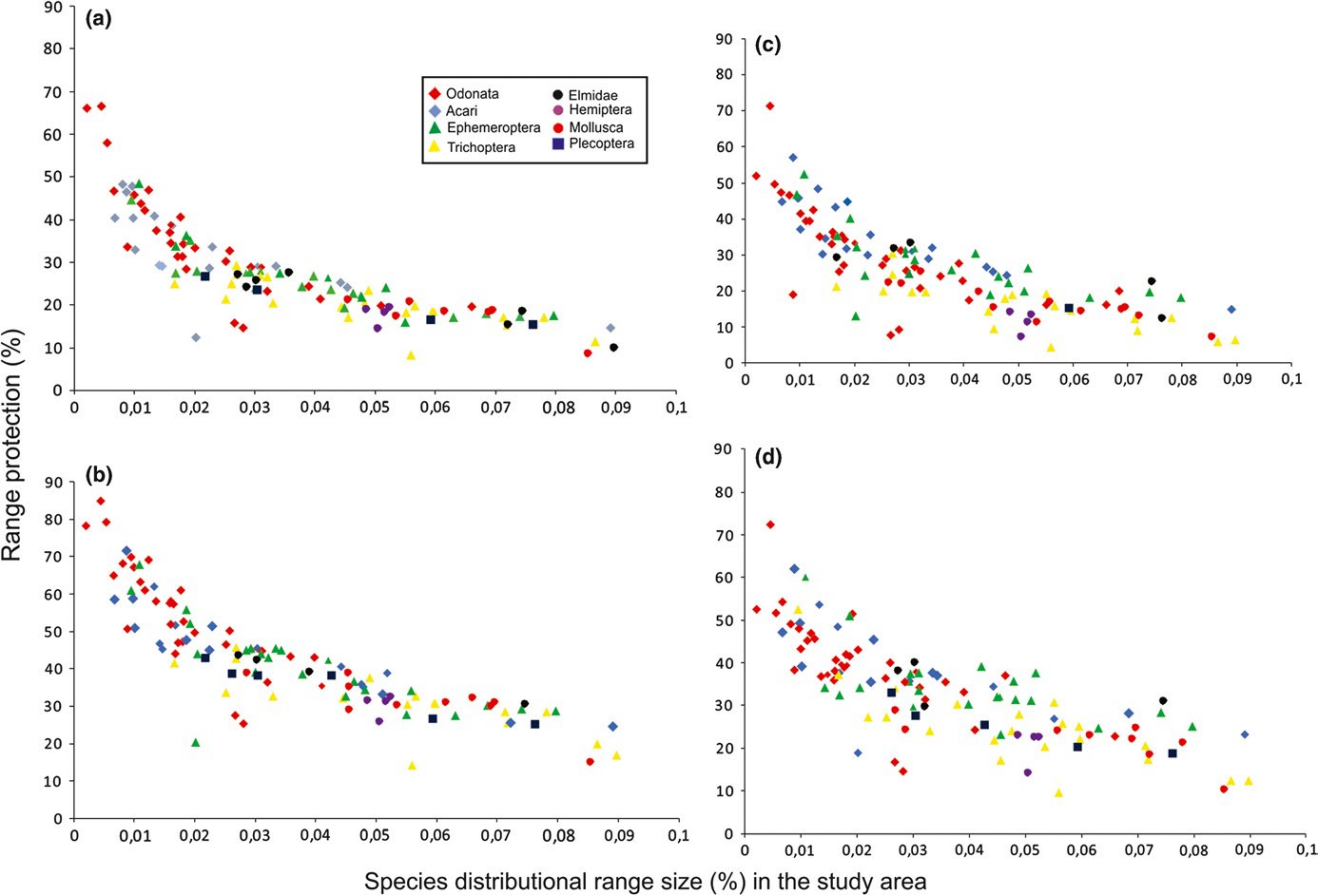
Comparisons between the distributional range protection for species (*y* axis) versus distributional range size (*x* axis) in two scenarios analyses (A2 and A3). (a) Analysis 2 considering 10% of priority of the total area; (b) analysis 2 considering 17% of priority area of the total area. (c) Analysis 3 considering 10% of priority of the total area; (d) analysis 3 considering 17% of priority of the total area

When we considered the sub‐basins with the 10% priority level, 25.64% of the average distribution of the macroinvertebrates were located in these units (Figure 4a). Some species with a smaller distribution range size will be more protected than species with a higher range size (Appendix S3). If 10% of the total area is considered, only eight species will be protected: Bivalvia (*Pisidium omaguaca*), Odonata (*Macrothemis hahneli* and *Andina griongarrisoni*); Trichoptera (*Atopsyche* (*Atopsaura*) *yunguensis*,* Smicridea* (*Rhyacophylax*) *chicoana*,* Marilia elongata* and *Anomalocosmoecus argentinicus* and Hemiptera (*Eurygerris fucinervis*).

If the analysis considers the units with 17% priority, 32.59% of the average distribution of the species considered will be protected (Figure 4b). About 20‐50% of almost all species distribution will be protected (Appendix S3). The species most protected will be *Micrathyria hypodidyma* with 84.9%, and *Erythrodiplax umbrata* (Odonata) with 78%, and *Hygrobatella multiacetabulata* (Acari) with 71%.

When taking land use of the respective areas into account (A3), similar basins were identified as top priority, but additional basins were suggested: San Francisco river (Jujuy), upper part of Juramento river (Catamarca, Salta and Tucumán), Falda Oriental de Ambato basin, and Salinas Grandes basin (Figure 3c). In the case of the San Francisco river, the sub‐basins with the top rank of priority conservation were lower San Francisco, Quebrada de Humahuaca, Ledesma, and Mojotoro‐Lavallén. In the case of the upper part of Juramento river basin, only two sub‐basins had the top rank of priority: Conchas‐Guachipas river, Santa María, and Calchaquí river.

When the analysis considered the sub‐basins with 10% priority, 26.80% of the average distribution of the macroinvertebrates will be located in these units (Figure 4c). The species with smaller distribution range size will be more protected than species with higher range size (Appendix S3). The species with less than 2% of distribution range size will be the most protected species, with 25‐66.6%. If the analysis considers the units with 17% of priority, 41.7% of the average distribution of the species considered will be protected (Figure 4d).

## 4 DISCUSSION

### 4.1 Protected areas

We did not find a correspondence between the proposed priority areas of conservation for aquatic macroinvertebrates and those areas currently within the protected areas system in the northwest of Argentina. The lack of consideration of invertebrate biodiversity in conservation planning is a global problem; more than a million species of invertebrates are known but only 3,500 species of arthropods are protected in the world (Baillie, Hilton‐Taylor, & Stuart, 2004; Brooks et al., 2004, 2006). This tendency declines if the aquatic systems are considered; only a single project of conservation priorities explicitly included aquatic systems (Olson & Dinerstein, 1998). Furthermore, it was reported that the lowest percentage (0.1%) of publications on the conservation of aquatic insects (Contador, Kennedy, & Rozzi, 2012) for South America, comparing it with the rest of the world.

Other studies carried out in Argentina have also clearly shown that protected areas are ineffective or insufficient for the preservation of biological diversity (Arzamendia & Giraudo, 2004; Nori et al., 2013; Tognelli et al., 2011). Although the protected areas are partial solutions to habitat degradation, they are also cornerstones of conservation (Margules & Pressey, 2000). At the same time, it is necessary that well‐planned protected areas represent both aquatic and terrestrial ecosystems as they are not independent units (Wuethrich, 2000).

### 4.2 Biodiversity conservation

Both analyses, A2 and A3, obtained a protection between 26% and 27% of the average distribution of the species considered when 10% of the planning units were protected (as proposed by CBD). When 17% of the planning units were protected, these ranges were increased to 33%–42%. Several authors have stated that it is impossible to establish a single universal target for the conservation for any kind of ecosystems (Rodrigues & Gaston, 2001). Worse still it is not clear which target is required to safeguard an individual species (Pidgeon, Rivera, Martinuzzi, Politi, & Bateman, 2015). Regarding vertebrates, Rodrigues et al. (2004) considered that conserving 10% of the land surface was enough if the species is widely distributed, and that this percentage must be higher, 70%–100%, if the species has a restricted distribution (Kukkala & Moilanen, 2012; Tognelli et al., 2011). However, Howard et al. (1998) and Kerr (1997) sustained that the minimum requirements to conserve the diversity of vertebrates will not necessarily be an effective umbrella for biodiversity in general, because many other more diverse groups (including plants and invertebrates) are expected to require considerably larger areas to be fully represented.

In addition, in designing efficient and effective conservation areas for freshwater, it is crucial to consider their connectivity (Hermoso, Kennard, & Linke, 2012). The area networks in freshwaters allow for genetic flow between populations. In this case, not only are longitudinal connections important, but also lateral connections, surface/groundwater, and spatial hierarchies of fluvial ecosystems (Fausch, Torgersen, Baxter, & Li, 2002; Turak & Linke, 2011).

Due to their high sensitivity to environmental stress (Contador et al., 2012), aquatic ecosystems need special protection and 10% or even 17% of land conservation is insufficient for these systems. This is especially true if the future extinction rate of freshwater animals becomes almost five times greater than for terrestrial animals (Ricciardi & Rasmussen, 1999).

### 4.3 Conservation prioritization areas

The A2 and A3 analyses identified the upper part of Bermejo river and Salí‐Dulce basins as priority basins for conservation. Two additional basins, the upper part of Juramento river and San Francisco river, were added with the analysis of the Balancing Alternative Land Uses (A3). These priority river basins are in line with native forest law which is currently enforced in Argentina. This law promotes the protection of forests with native species and the riparian forests. This fact reinforces the necessity to conserve not only the water course but also the riparian forests in order to preserve both aquatic and terrestrial ecosystems (Arzamendia & Giraudo, 2012; Mesa, Reynaga, Correa, & Sirombra, 2013). Moreover, the protection of buffer zones or riparian vegetation was found to be of prime importance as impact‐filterers in unprotected areas or in agricultural lands (Diebel, Maxted, Robertson, Han, & Vander Zanden, 2009). The buffer areas of 50 m on each margin are the minimum necessary to reduce significantly the input of nutrients and agrochemicals to the rivers (Stickler, Nepstad, Azevedo, & McGrath, 2013). Argentinian laws protect riverside vegetation, but recently the width of this buffer area was significantly reduced from 35 to 15 m at each margin of navigable rivers (Infojus, 2014), which has been found to be insufficient in similar ecosystems from Brazil (Nagy et al., 2015). Additionally, the control of this buffer area is not enforced at all in any region of Argentina, so its actual value as a conservation tool is null.

The Bermejo, Salí‐Dulce, Juramento, and San Francisco basins are subjected to strong anthropic pressures. The main cities and industries are located here, as well as the development of agriculture and livestock. This results in conflicts of interest, and the biodiversity value is not taken into account. Saunders, Meeuwig, and Vincent (2002) recommended whole‐catchment management in order to conserve freshwater habitats and species. They proposed alternatives where the protection of the entire basin is not feasible. One possible alternative they may apply here is the creation of multiple‐use modules (NUMs) (Noss & Harris, 1986). A NUM consists of a central, well‐protected core surrounded by a series of buffer zones in which varying human activities are permitted. Even more, this alternative could be combined with the theory of River Continuum and the well‐protected core could be located at the headwaters of the river basins (Saunders et al., 2002) guaranteeing a good water discharge rate throughout the entire year in these monsoonal climates. Also, a good habitat quality in the headwaters will provide food resources (coarse particulate organic matter, CPOM) to and seedling recruitments (drift, aerial dispersion) of aquatic species. Nevertheless, the heterogeneous nature of the landscape in NW Argentina (four large ecoregions may be represented in a sole watershed) needs a strong complement to the NUM′s: the buffer areas of marginal vegetation. These areas must be preserved and restored through the entire river continuum, not only in the headwaters, but also in the more human‐pressed piedmont and lowlands. Productive areas in many countries are benefiting from restored marginal vegetation, even at low or inexistent costs (promoting natural succession), resulting in increased water quality, animal and plant production, and human health (Tognetti, Chaneton, Omacini, Trebino, & León, 2010). In this way, conservation planning of freshwaters includes not only the freshwater systems but some elements of terrestrial biodiversity, recognizing the necessity to integrate both systems (Amis, Rouget, Lotter, & Day, 2009).

The paradigm of delimiting conservation units to secure biodiversity is based on the grounds of the dominance of humankind over nature. The occurrence today of completely modified ecosystems draws our attention to we are relating to the biosphere as a species. We consider that the protection of marginal vegetation on every reach of every water body, as a “belt NUM” around these ecosystems, as indicated by the present study, will aid in a conservation paradigm shift that many human groups and some nations are promoting around the World. Only when the man feels part of the environment will he seek to preserve it, changing the paradigm of dominance to that of protector of the environment.

## CONFLICT OF INTEREST

None declared.
