# Erratum

**DOI:** 10.1002/ece3.3434

**Published:** 2017-09-20

This article corrects:

The role of macroinvertebrates for conservation of freshwater systems

Volume 7, Issue 14, 5502‐5513, Article first published online: 15 June 2017

Nieto C., Ovando XMC, Loyola R, et al. (2017) The role of macroinvertebrates for conservation of freshwater systems. Eco Evol. 2017; 7:5502‐5513. https://doi.org/10.1002/ece3.3101

In the article “The role of macroinvertebrates for conservation of freshwater systems,” the authors wish to note that Figure 3 appeared incorrectly. Please find the correct figure below.

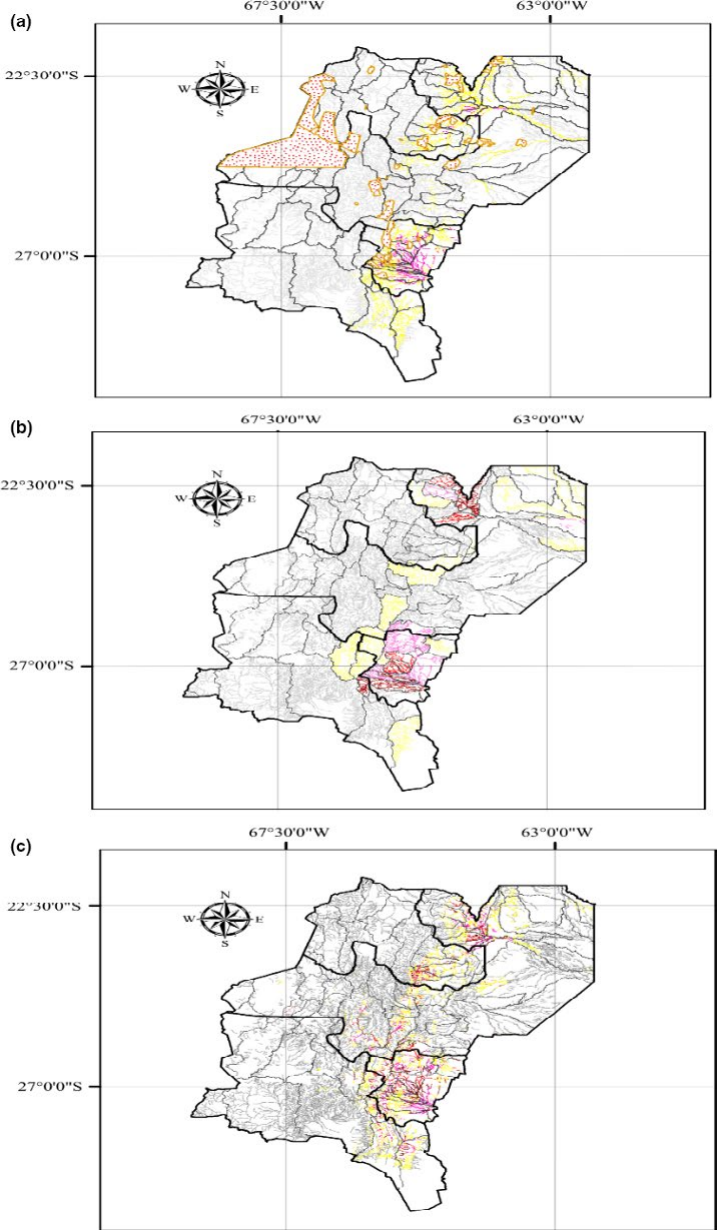

We would like to apologize for any inconvenience this has caused.

