# Supplementary material for: The role of macroinvertebrates for conservation of freshwater systems
Source: Ecol Evol. 2017 Jun 15;7(14):5502–13. doi: 10.1002/ece3.3101 (PMC5528230; doi:10.1002/ece3.3101)
Supplement: Supplementary file 1 [file ECE3-7-5502-s001.docx]

Appendix 1. Total species included in the analysis.

| **Taxon** | **Species** | **Number of records** | **Model performance obtained from Maxent analysis:**  **AUC:** Area Under the Curve measure | **Main Variable to contribute in the Maxent Model of each species** |
| --- | --- | --- | --- | --- |
| **Insects** |  |  |  |  |
| **Elmidae** | *Austrelemis argentinensis* | 20 | 0.894 | Bioclimatic 12 |
|  | *Austremils robustus* | 8 | 0.846 | Bioclimatic 3 |
|  | *Austrelmis tafi* | 4 | 0.757 | Bioclimatic 2 |
|  | *Cylloepus calchaqui* | 5 | 0.837 | Bioclimatic 13 |
|  | *Heterelmis rufus* | 12 | 0.809 | Altitude |
|  | *Macrelmis isis* | 24 | 0.901 | Bioclimatic 12 |
|  | *Macrelmis tucumanensis* | 16 | 0.861 | Bioclimatic 2 |
|  | *Austrolimnius formosus* |  |  |  |
|  | *Cylloepus vianai* |  |  | ** |
|  | *Macrelmis saltensis* |  |  |  |
|  | *Hexanchorus saltensis* |  |  |  |
|  | *Phanocerus hintoni* |  |  | ** |
| **Ephemeroptera** | *Americabaetis alphus* | 37 | 0.897 | Soil |
|  | *Andesiops peruvianus* | 18 | 0.907 | Bioclimatic 6 |
|  | *Apobaetis insolitus* | 2 |  | * |
|  | *Apobaetis niger* | 4 | 0.690******* | Bioclimatic 13 |
|  | *Atopophlebia flowersi* | 2 |  | * |
|  | *Baetodes cochunaensis* | 11 | 0.770 | Bioclimatic 13 |
|  | *Baetodes copiosus* | 12 | 0.886 | Bioclimatic 2 |
|  | *Baetodes huaico* | 41 | 0.916 | Bioclimatic 2 |
|  | *Caenis argentina* | 7 | 0.982 | Soil |
|  | *Caenis dominguezi* | 7 | 0.883 | Bioclimatic 3 |
|  | *Caenis ludicra* | 32 | 0.886 | Bioclimatic 12 |
|  | *Callibaetis dominguezi* | 1 |  | * |
|  | *Camelobaetidius penai* | 67 | 0.901 | Soil |
|  | *Cloeodes barituensis* | 6 | 0.753 | Bioclimatic 13 |
|  | *Cloeodes incus* | 2 |  | * |
|  | *Euthyplocia hecuba* | 4 | 0.997 | Bioclimatic 13 |
|  | *Farrodes yungaensis* | 18 | 0.890 | Bioclimatic 12 |
|  | *Guajirolus queremba* | 6 | 0.964 | Altitude |
|  | *Hagenulopsis lipeo* | 4 |  | * |
|  | *Haplohyphes baritu* | 31 | 0.919 | Bioclimatic 2 |
|  | *Leptohyphes eximius* | 97 | 0.900 | Bioclimatic 12 |
|  | *Lumahyphes guacra* | 15 | 0.947 | Altitude |
|  | *Massartellopsis irrarrazavali* | 7 | 0.975 | Bioclimatic 2 |
|  | *Nanomis galera* | 14 | 0.959 | Bioclimatic 13 |
|  | *Thraulodes cochunaensis* | 37 | 0.874 | Bioclimatic 12 |
|  | *Thraulodes consortis* | 41 | 0.895 | Bioclimatic 12 |
|  | *Thraulodes liminaris* | 6 | 0.825 | Bioclimatic 13 |
|  | *Tortopsis obscuripennis* | 5 | 0.944 | Bioclimatic 13 |
|  | *Tortopsis sarae* | 7 | 0.918 | Altitude |
|  | *Tricorythodes hiemalis* | 6 | 0.946 | Bioclimatic 13 |
|  | *Tricorythodes popayanicus* | 54 | 0.879 | Bioclimatic 12 |
|  | *Tricorythodes quizeri* | 6 | 0.962 | Altitude |
|  | *Varipes minutus* | 6 | 0.876 | Bioclimatic 13 |
|  | *Varipes singuil* | 11 | 0.860 | Altitude |
|  | *Yaurina mota* | 1 |  | * |
| **Hemiptera** | *Ambrysus bergi* |  |  | ** |
|  | *Ambrysus fucatus* |  |  | ** |
|  | *Ambrysus gemignani* |  |  | ** |
|  | *Ambrysus kolla* | 4 | 0.841 | Bioclimatic13 |
|  | *Eurygerris fucinervis* | 5 | 0.915 | Bioclimatic13 |
|  | *Limnocoris ovatulus* | 9 | 0.909 | Bioclimatic13 |
| **Lepidoptera** | *Nymphula effetanalis* | 4 | 0.943 | Bioclimatic13 |
| **Megaloptera** | *Corydalus armatus* | 26 | 0.796 | Bioclimatic13 |
|  | *Corydalus longicornis* | 3 |  | * |
|  | *Corydalus peruvianus* | 4 |  | ** |
|  | *Corydalus primitivus* | 37 | 0.878 | Altitude |
| **Odonata** | *Acanthagrion floridense* | 19 | 0.900 | Bioclimatic 12 |
|  | *Acanthagrion lancea* | 8 | 0.927 | Bioclimatic 12 |
|  | *Andinagrion garrisoni* | 4 | 0.983 | Bioclimatic 19 |
|  | *Argia joergenseni* | 14 | 0.885 | Bioclimatic 13 |
|  | *Argia jujuya* | 8 | 0.906 | Bioclimatic 2 |
|  | *Cannaphila vibex* | 8 | 0.976 | Bioclimatic 2 |
|  | *Dasythemis mincki* |  |  |  |
|  | *Diastatops intensa* |  |  |  |
|  | *Dythemis multipunctata* | 5 | 0.964 | Bioclimatic 12 |
|  | *Erythemis attala* | 8 | 0.912 | Bioclimatic 13 |
|  | *Erythrodiplax atroterminata* |  |  |  |
|  | *Erythrodiplax media* | 7 | 0.922 | Bioclimatic 12 |
|  | *Erythrodiplax nigricans* |  |  |  |
|  | *Erythrodiplax umbrata* | 6 | 0.996 | Soil |
|  | *Gynacanta adela* | 4 | 0.844 | Bioclimatic 13 |
|  | *Hetaerina rosea* | 8 | 0.957 | Soil |
|  | *Ischnura capreolus* | 12 | 0.948 | Bioclimatic 2 |
|  | *Ischnura fluviatilis* | 8 | 0.913 | Bioclimatic 3 |
|  | *Ischnura ultima* | 8 | 0.970 | Bioclimatic 2 |
|  | *Macrothemis hahneli* | 7 | 0.929 | Bioclimatic 13 |
|  | *Macrothemis imitans* | 9 | 0.922 | Bioclimatic 2 |
|  | *Micrathyria hypodidyma* | 6 | 0.984 | Bioclimatic 12 |
|  | *Micrathyria longifasciata* | 2 |  | * |
|  | *Micrathyria venezuelae* |  |  |  |
|  | *Mnesarete grisea* | 14 | 0.954 | Bioclimatic 2 |
|  | *Neoneura confundens* | 4 | 0.941 | Altitude |
|  | *Orthemis discolor* | 5 | 0.992 | Bioclimatic 2 |
|  | *Orthemis nodiplaga* | 4 | 0.975 | Altitude |
|  | *Oxyagrion ablutum* | 26 | 0.911 | Bioclimatic 2 |
|  | *Pantala flavescens* | 5 | 0.968 | Soil |
|  | *Perithemis mooma* | 6 | 0.939 | Altitude |
|  | *Phyllocycla argentina* | 5 | 0.983 | Altitude |
|  | *Progomphus complicatus* | 6 | 0.947 | Bioclimatic 14 |
|  | *Progomphus kimminsi* | 3 |  | * |
|  | *Progomphus phyllocromus* | 4 | 0.979 | Bioclimatic 2 |
|  | *Remartinia luteipennis* | 3 |  | * |
|  | *Rhionaeschna planaltica* | 12 | 0.970 | Bioclimatic 2 |
|  | *Rhionaeschna vigintipunctata* | 11 | 0.973 | Bioclimatic 2 |
|  | *Teinopodagrion meridionale* | 5 | 0.966 | Bioclimatic 2 |
| **Plecoptera** | *Anacroneuria saltensis* | 2 |  | * |
|  | *Claudioperna tigrina* | 18 | 0.827 | Bioclimatic2 |
| **Trichoptera** | *Alisotrichia benji* | 1 |  | * |
|  | *Anchitrichia trifurcata* | 1 |  | * |
|  | *Anomalocosmoecus argentinicus* | 7 | 0.830 | Bioclimatic 17 |
|  | *Atopsyche (Atopsaura) spinosa* | 14 | 0.725 | Bioclimatic 13 |
|  | *Atopsyche (Atopsaura) yunguensis* | 5 | 0.853 | Bioclimatic 18 |
|  | *Banyallarga argentinica* | 23 | 0.781 | Bioclimatic 18 |
|  | *Cailloma lucidula* | 8 | 0.989 | Bioclimatic 3 |
|  | *Chimarra argentinica* | 8 | 0.860 | Bioclimatic 13 |
|  | *Helicopsyche turbida* | 4 | 0.833 | Bioclimatic 13 |
|  | *Hydroptila argentinica* | 17 | 0.688 | Bioclimatic 13 |
|  | *Hydroptila bidens* | 4 | 0.812 | Bioclimatic 12 |
|  | *Hydroptila catamarcensis* | 5 | 0.930 | Bioclimatic 3 |
|  | *Hydroptila coscaroni* | 1 |  | * |
|  | *Ithytrichia ferni* | 4 |  | * |
|  | *Leptonema boliviense boliviense* | 10 | 0.845 | Bioclimatic 13 |
|  | *Leptonema boliviense plumosum* | 2 |  | * |
|  | *Leucotrichia alisensis* | 1 |  | * |
|  | *Leucotrichia yungarum* | 1 |  | * |
|  | *Marilia cinerea* | 16 | 0.799 | Bioclimatic 6 |
|  | *Marilia elongata* | 5 | 0.962 | Bioclimatic 18 |
|  | *Merionoptila wygodzinskyi* | 2 |  | * |
|  | *Metrichia neotropicalis* | 5 | 0.943 | Bioclimatic 13 |
|  | *Mortoniella wygodzinskii* | 6 | 0.793 | Bioclimatic 13 |
|  | *Neotrichia sala* | 1 |  | * |
|  | *Oxyethira baritu* | 1 |  | * |
|  | *Polycentropus jorgenseni* | 7 | 0.934 | Bioclimatic 3 |
|  | *Smicridea (Rhyacophylax) atrobasis* | 5 | 0.825 | Altitude |
|  | *Smicridea (Rhyacophylax) bifida* | 1 |  | * |
|  | *Smicridea (Rhyacophylax) elisae* | 1 |  | * |
|  | *Smicridea (Rhyacophylax) peruana* | 14 | 0.702 | Bioclimatic 6 |
|  | *Smicridea (Smicridea) chicoana* | 6 | 0.768 | Bioclimatic 13 |
|  | *Xiphocentron caenina* | 1 |  | * |
|  | *Xiphocentron pintada* | 1 |  | * |
| **Non insects** |  |  |  |  |
| **Acari** | *Atractidella porophora* | 7 | 0.774 | Bioclimatic 13 |
|  | *Atractides brasiliensis* |  |  |  |
|  | *Atractides sinuatipes* | 28 | 0.957 | Bioclimatic 2 |
|  | *Axonopsella argentinensis* |  |  |  |
|  | *Clathrosperchon punctatus* | 26 | 0.944 | Bioclimatic 2 |
|  | *Corticacarus brassanus* | 10 | 0.724******* | Bioclimatic 18 |
|  | *Corticacarus smithi* | 13 | 0.898 | Bioclimatic 3 |
|  | *Dodecabates dodecaporus* | 20 | 0.950 | Bioclimatic 2 |
|  | *Hygrobatella coriacea* | 3 |  | * |
|  | *Hygrobatella multiacetabulata* | 4 | 0.992 | Bioclimatic 3 |
|  | *Hygrobates ampliatus* |  |  |  |
|  | *Hygrobates plebejus* | 8 | 0.893 | Bioclimatic 13 |
|  | *Protolimnesia interstitialis* | 9 | 0.936 | Bioclimatic 13 |
|  | *Protolimnesia setifera* | 15 | 0.934 | Bioclimatic 2 |
|  | *Protolimnesia sorpresa* | 1 |  | * |
|  | *Rhyncholimnochares dipersiai* | 1 |  | * |
|  | *Rhyncholimnochares expansiseta* | 22 | 0.966 | Bioclimatic 2 |
|  | *Sperchon neotropicus* | 2 |  | * |
|  | *Stygalbiella tucumanensis* | 13 | 0.900 | Bioclimatic 2 |
|  | *Tetrahygrobatella argentinensis* | 14 | 0.935 | Bioclimatic 2 |
|  | *Tetrahygrobatella bovala* | 11 | 0.942 | Bioclimatic 3 |
|  | *Torrenticola columbiana* | 44 | 0.945 | Bioclimatic 12 |
| **Bivalvia** | *Pisidium chiquitanum* | 6 | 0.862 | Bioclimatic13 |
|  | *Pisidium ocloya* | 11 | 0.717 | Bioclimatic13 |
|  | *Pisidium omaguaca* | 7 | 0.665******* | Bioclimatic3 |
|  | *Psidium chicha* |  |  | ** |
| **Gastropoda** | *Biomphalaria orbignyi* | 4 | 0.666 ******* | Bioclimatic3 |
|  | *Biomphalaria peregrina* | 15 | 0.829 | Soil |
|  | *Biomphalaria tenagophila* | 35 | 0.929 | Bioclimatic12 |
|  | *Drepanotrema depressissimum* | 11 | 0.821 | Bioclimatic13 |
|  | *Drepanotrema kermatoides* | 11 | 0.751 | Altitude |
|  | *Drepanotrema lucidum* | 4 | 0.929 | Bioclimatic13 |
|  | *Drepanotrema nordestense* |  |  | */** |
|  | *Galba viator* | 38 | 0.891 | Bioclimatic12 |
|  | *Gundlachia radiata* | 9 | 0.894 | Bioclimatic13 |
|  | *Omalonyx convexus* | 12 | 0.810 | Rivers/streams |
|  | *Stenophysa minor* | 17 | 0.811 | Altitude |
|  | *Uncancylus concentricus* | 28 | 0.902 | Bioclimatic13 |

|  | Species Modeled |
| --- | --- |

|  | Species not Modeled |
| --- | --- |

*SSI: species of special interest

**Models did not obtain p < 0.05 according to the Jacknife validation

***Models with p < 0.05 according to the Jacknife validation
