# Supplementary material for: The role of macroinvertebrates for conservation of freshwater systems
Source: Ecol Evol. 2017 Jun 15;7(14):5502–13. doi: 10.1002/ece3.3101 (PMC5528230; doi:10.1002/ece3.3101)
Supplement: Supplementary file 2 [file ECE3-7-5502-s002.docx]

Appendix 2. Variables used in this study. Bioclimatic 1- Bioclimatic 19 from the WorldClim data base.

| **Variables** | **Description** |
| --- | --- |
| Bioclimatic 1 | Annual mean temperature |
| Bioclimatic 2 | Mean diurnal range (monthly mean, Tº max- Tº min) |
| Bioclimatic 3 | Isothermality (bio2/bio7) x 100 |
| Bioclimatic 4 | Temperature seasonality (standard deviation x 100) |
| Bioclimatic 5 | Maximum temperature of warmest month |
| Bioclimatic 6 | Minimum temperature of coldest month |
| Bioclimatic 7 | Temperature annual range (bio 5- bio 6) |
| Bioclimatic 8 | Mean Temperature of wettest quarter |
| Bioclimatic 9 | Mean Temperature of driest quarter |
| Bioclimatic 10 | Mean temperature of the warmest quarter |
| Bioclimatic 11 | Mean Temperature of coldest quarter |
| Bioclimatic 12 | Annual precipitation |
| Bioclimatic 13 | Precipitation of wettest month |
| Bioclimatic 14 | Precipitation of driest month |
| Bioclimatic 15 | Precipitation seasonality (coefficient of variation) |
| Bioclimatic 16 | Precipitation of wettest quarter |
| Bioclimatic 17 | Precipitation of driest quarter |
| Bioclimatic 18 | Precipitation of the warmest quarter |
| Bioclimatic 19 | Precipitation of the coldest quarter |
| Variable 20 | Soil |
| Variable 21 | Rivers/ streams |
| Variable 22 | Altitude |
