# Supplementary material for: The role of macroinvertebrates for conservation of freshwater systems
Source: Ecol Evol. 2017 Jun 15;7(14):5502–13. doi: 10.1002/ece3.3101 (PMC5528230; doi:10.1002/ece3.3101)
Supplement: Supplementary file 3 [file ECE3-7-5502-s003.docx]

Appendix 3: List of species included in the Zonation analyses: A2 and A3, and with 10% or 17% of priority of each sub-basin. Values represent the percentage of protection for each species.

|  | **A2** |  | **A3** |  |
| --- | --- | --- | --- | --- |
|  | Sub-basin with 10% of priority | Sub-basin with 17% of priority | Sub-basin with 10% of priority | Sub-basin with 10% of priority |
| ***Species*** |  |  |  |  |
| *Atractidella porophora* | 30 | 35.6 | 28.7 | 45.1 |
| *Atractides sinuatipes* | 37 | 39.1 | 32.9 | 50.9 |
| *Clathrosperchon punctatus* | 34.6 | 37.2 | 29 | 45.3 |
| *Corticacarus brassanus* | 14.9 | 23.2 | 14.7 | 24.7 |
| *Corticacarus smithi* | 26.6 | 34.5 | 25.3 | 40.5 |
| *Dodecabates dodecaporus* | 48.4 | 53.5 | 40.8 | 62 |
| *Hygrobatella multiacetabulata* | 57 | 62 | 46.4 | 71.6 |
| *Hygrobates plebejus* | 30.8 | 37.8 | 29 | 45.6 |
| *Protolimnesia interstitialis* | 25.2 | 32.1 | 24.1 | 38.9 |
| *Protolimnesia setifera* | 45.7 | 49.2 | 40.4 | 58.8 |
| *Rhyncholimnochares expansiseta* | 44.8 | 47.1 | 40.3 | 58.6 |
| *Stygalbiella tucumanensis* | 29 | 37.7 | 29 | 45.4 |
| *Tetrahygrobatella argentinensis* | 43.2 | 48.5 | 38.5 | 57.4 |
| *Tetrahygrobatella bovala* | 35.6 | 45.5 | 33.6 | 51.4 |
| *Torrenticola columbiana* | 30.2 | 34.2 | 29.4 | 46.7 |
| *Acanthagrion floridense* | 24 | 35.4 | 27.5 | 43.3 |
| *Acanthagrion lancea* | 35 | 36.8 | 37.4 | 58.1 |
| *Andinagrion garrisoni* | 9.2 | 14.5 | 14.7 | 25.4 |
| *Argia joergenseni* | 22.7 | 30.3 | 26.9 | 43 |
| *Argia jujuya* | 27.1 | 36.4 | 30.3 | 46.6 |
| *Cannaphila vibex* | 39.3 | 47 | 42.1 | 61 |
| *Dythemismulti punctata* | 27.2 | 42 | 31.4 | 47.1 |
| *Erythemis attala* | 34.9 | 40.6 | 38.7 | 58.2 |
| *Erythrodiplax media* | 29 | 40 | 32.6 | 50.2 |
| *Erythrodiplax umbrata* | 51.8 | 52.6 | 66 | 78.3 |
| *Gynacantha adela* | 17.3 | 24.3 | 21.5 | 35.4 |
| *Hetaerina rosea* | 20.8 | 31.3 | 23.2 | 36.5 |
| *Ischnura capreolus* | 35.5 | 38.7 | 40.5 | 61.1 |
| *Ischnura fluviatilis* | 16 | 22.8 | 19.7 | 32.2 |
| *Ischnura ultima* | 26.5 | 34.1 | 28.8 | 44.7 |
| *Macrothemis hahneli* | 7.5 | 16.8 | 15.8 | 27.5 |
| *Macrothemis imitans* | 25.5 | 37.4 | 28.8 | 44.9 |
| *Micrathyria hypodidyma* | 71.3 | 72.3 | 66.6 | 84.9 |
| *Mnesarete grisea* | 34.3 | 39.3 | 34.2 | 52.6 |
| *Neoneura confundens* | 18.8 | 38.2 | 33.5 | 50.6 |
| *Orthemis discolor* | 41.3 | 43.2 | 45.8 | 67.2 |
| *Orthemis nodiplaga* | 46.5 | 49 | 48.2 | 68.2 |
| *Oxyagrion ablutum* | 36.2 | 38.1 | 34.4 | 52 |
| *Pantala flavescens* | 25.2 | 39.6 | 31.4 | 46.9 |
| *Perithemis mooma* | 32.9 | 36 | 37 | 57.5 |
| *Phyllocycla argentina* | 49.6 | 51.7 | 57.9 | 79.1 |
| *Progomphus complicatus* | 33.3 | 43.1 | 33.3 | 49.8 |
| *Progomphus phyllocromus* | 42.4 | 45.6 | 47 | 69.2 |
| *Rhionaeschna planaltica* | 39.5 | 45.2 | 43.7 | 63.1 |
| *Rhionaeschna vigintipunctata* | 47.3 | 54.2 | 46.6 | 64.8 |
| *Teinopodagrion meridionale* | 45.5 | 47.9 | 47.9 | 69.9 |
| *Anomalocosmoecus argentinicus* | 4.4 | 9.7 | 8.3 | 14.4 |
| *Atopsyche (Atopsaura) yunguensis* | 6.3 | 12.4 | 9.9 | 17 |
| *Banyallarga argentinica* | 14.2 | 21.9 | 19.5 | 32.3 |
| *Cailloma lucidula* | 20 | 27.3 | 21.3 | 33.7 |
| *Chimarra argentinica* | 12.4 | 21.2 | 17.1 | 28.6 |
| *Helicopsyche turbida* | 19 | 27.9 | 23.4 | 37.7 |
| *Hydroptila bidens* | 14.2 | 25.2 | 18.6 | 31 |
| *Hydroptila catamarcensis* | 21.1 | 37.2 | 25.1 | 41.6 |
| *Leptonema boliviense boliviense* | 9.4 | 17.1 | 17.2 | 29 |
| *Marilia cinerea* | 14.4 | 22.1 | 18.4 | 30.8 |
| *Marilia elongata* | 8.9 | 17.3 | 15 | 25.6 |
| *Metrichia neotropicalis* | 17.8 | 24.1 | 22.4 | 36 |
| *Mortoniella wygodzinskii* | 12.3 | 20.6 | 17.1 | 28.6 |
| *Polycentropus jorgenseni* | 24.4 | 28.8 | 29.3 | 45.8 |
| *Smicridea (Rhyacophylax) atrobasis* | 15.7 | 25.7 | 19.9 | 32.8 |
| *Smicridea (Rhyacophylax) chicoana* | 5.8 | 12.5 | 11.5 | 20 |
| *Americabaetis alphus* | 30.4 | 33.9 | 27 | 42.8 |
| *Andesiops peruvianus* | 19.7 | 24.1 | 20.5 | 32.7 |
| *Baetodes cochunaensis* | 18.1 | 25.2 | 17.5 | 28.8 |
| *Baetodes copiosus* | 25.9 | 30.3 | 24.4 | 38.5 |
| *Baetodes huaico* | 32.1 | 34.1 | 27.9 | 44.1 |
| *Caenis argentina* | 46.9 | 52.6 | 44.6 | 60.9 |
| *Caenis dominguezi* | 23.9 | 36.9 | 22.7 | 36.7 |
| *Caenis ludicra* | 30.2 | 35.5 | 27.6 | 45.6 |
| *Camelobaetidius penai* | 31.3 | 35.5 | 27.4 | 45 |
| *Cloeodes barituensis* | 22.3 | 31.3 | 20.7 | 34.5 |
| *Euthyplocia hecuba* | 31.7 | 41.5 | 28.4 | 47.8 |
| *Farrodes yungaensis* | 30.4 | 39.1 | 26.4 | 42.4 |
| *Guajirolus queremba* | 40.2 | 51.5 | 35.2 | 52.1 |
| *Haplohyphes baritu* | 29.2 | 32.4 | 27.6 | 44.1 |
| *Leptohyphes eximius* | 31.5 | 35.7 | 27.7 | 45.4 |
| *Lumahyphes guacra* | 44.8 | 51.1 | 36.3 | 55.9 |
| *Massartellopsis irrarrazavali* | 18.1 | 24.8 | 17.1 | 27.5 |
| *Nanomis galera* | 35.2 | 37.6 | 33.9 | 51.6 |
| *Thraulodes cochunaensis* | 32 | 37.1 | 27.4 | 44.9 |
| *Thraulodes consortis* | 28.7 | 33.6 | 28 | 44.5 |
| *Thraulodes liminaris* | 16.1 | 26.9 | 16 | 27.7 |
| *Tortopsis obscuripennis* | 19.9 | 31.2 | 19.9 | 33.2 |
| *Tortopsis sarae* | 24.2 | 35.7 | 21.9 | 35.3 |
| *Tricorythodes hiemalis* | 18.8 | 32.1 | 19.4 | 32.7 |
| *Tricorythodes popayanicus* | 31.6 | 37.6 | 26.9 | 44.1 |
| *Tricorythodes quizeri* | 52.5 | 60 | 48.4 | 67.8 |
| *Varipes minutus* | 19.8 | 28.2 | 18.1 | 30.2 |
| *Varipes singuil* | 26.4 | 37.7 | 24.1 | 38.8 |
| *Austrelemis argentinensis* | 24.9 | 29.6 | 23.8 | 39 |
| *Austermils robustus* | 19.7 | 28.3 | 17.4 | 29.2 |
| *Austrelmis tafi* | 13.1 | 18.8 | 12.3 | 20.4 |
| *Cylloepus calchaqui* | 33.3 | 40 | 25.7 | 42.2 |
| *Heterelmis rufus* | 22.5 | 31 | 18.5 | 30.6 |
| *Macrelmis isis* | 31.7 | 38 | 27 | 43.6 |
| *Macrelmis tucumanensis* | 27.6 | 33.2 | 24.4 | 39.1 |
| *Ambrysus kolla* | 14.1 | 22.9 | 19 | 31.4 |
| *Eurygerris fucinervis* | 7 | 14.1 | 14.5 | 25.8 |
| *Limnocoris ovatulus* | 11.3 | 22.5 | 18.2 | 31.2 |
| *Nymphula effetanalis* | 13.3 | 22.5 | 19.3 | 32.4 |
| *Claudioperna tigrina* | 15 | 20.1 | 16.5 | 26.5 |
| *Corydalus armatus* | 19.7 | 25.4 | 23.7 | 38.1 |
| *Corydalus primitivus* | 19.7 | 27.6 | 23.5 | 38.1 |
| *Biomphalaria orbignyi* | 22.1 | 32.8 | 25 | 38.7 |
| *Biomphalaria peregrina* | 12.3 | 18.6 | 15.2 | 25.2 |
| *Biomphalaria tenagophila* | 24.3 | 27.3 | 26.5 | 42.7 |
| *Drepanotrema depressissimum* | 14.3 | 23 | 18.5 | 31 |
| *Drepanotrema kermatoides* | 15.2 | 24.8 | 18.8 | 31 |
| *Drepanotrema lucidum* | 11.3 | 20.5 | 17.3 | 30.3 |
| *Galba viator* | 21.9 | 24.2 | 24 | 38.9 |
| *Gundlachia radiata* | 15.3 | 23.1 | 21.1 | 35.3 |
| *Omalonyx convexus* | 14.8 | 22.2 | 18.3 | 30 |
| *Pisidium chiquitanum* | 13.1 | 18.5 | 15.4 | 25.6 |
| *Pisidium omaguaca* | 7 | 10.3 | 8.6 | 15.1 |
| *Stenophysa minor* | 16.8 | 24.1 | 20.7 | 34.1 |
| *Uncancylus concentricus* | 25.4 | 29.6 | 26.6 | 43 |
